# Elastography, a sensitive tool for the evaluation of neoadjuvant chemotherapy in patients with high-grade serous ovarian carcinoma

**DOI:** 10.3892/ol.2014.2346

**Published:** 2014-07-11

**Authors:** MENG XIE, XUYIN ZHANG, ZHAN JIA, YUNYUN REN, WENPING WANG

**Affiliations:** 1 Department of Ultrasound, Obstetrics and Gynecology Hospital, Fudan University, Shanghai 200090, P.R. China; 2 Department of Obstetrics and Gynecology, Obstetrics and Gynecology Hospital, Fudan University, Shanghai 200090, P.R. China; 3 Department of Ultrasound, Huadong Hospital, Fudan University, Shanghai 200050, P.R. China; 4 Department of Ultrasound, Zhongshan Hospital, Fudan University, Shanghai 200032, P.R. China

**Keywords:** elastography, high-grade serous ovarian cancer, neoadjuvant chemotherapy, sonography

## Abstract

The aim of the present study was to evaluate tumor stiffness by ultrasound elastography, which has the potential to provide additional information that is useful in predicting the response to neoadjuvant chemotherapy (NACT) in high-grade serous ovarian carcinoma (HGSC) patients. In total, 32 patients with International Federation of Gynecology and Obstetrics stage III and IV epithelial ovarian cancer treated with NACT underwent transvaginal and transabdominal sonography, followed by elastography and finally, by interval cytoreductive surgery. Histopathological analysis revealed 24 (75%) HGSCs. The mean elasticity score was statistically higher for the post-NACT lesions than for the pre-NACT lesions (3.13±0.57 vs. 2.04±0.51, respectively; P<0.001). The median elasticity score for the pre-NACT lesions on the four-point scale was 2, and the score for the post-NACT lesions was 4. Cases of post-NACT with scores of 3 and 4 had a higher optimal cytoreduction rate than cases with scores of 1 and 2 (93.8 vs. 25.0%, respectively; P<0.001). When the post-NACT elasticity scores of 3 and 4 were used for the prediction of optimal cytoreduction, elastography exhibited 88.2% sensitivity, 85.7% specificity, a 93.8% positive predictive value, a 75.0% negative predictive value and 87.5% accuracy. The results of the current study suggested that elastography is a sensitive tool for the evaluation of NACT in patients with HGSC and that it may aid gynecologists in choosing the optimal cytoreduction.

## Introduction

In general, the life-time risk of suffering from epithelial ovarian cancer (EOC) is ~1.5% in females. However, ~70% of females with EOC are diagnosed at advanced stages, for which the estimated long-term survival rate is ~10% (1). Neoadjuvant chemotherapy (NACT) prior to delayed primary or interval debulking surgery subsequent to an initial suboptimal surgery, followed by several periods of chemotherapy, have been suggested as substitutions to primary debulking surgery (2).

The golden criterion of EOC is the pathological diagnosis, which shows a complete or almost complete response to NACT (2). Therefore, accurate and non-invasive evaluation of the tumor response to adjuvant therapy is likely to be extremely useful. Until now, the efficacy of therapy has been evaluated using morphological changes as determined by imaging techniques, such as ultrasound, computed tomography (CT), magnetic resonance imaging (MRI) and positron emission tomography (PET) (3). These techniques rely on changes in the size of the mass to assess the tumor response to therapy. However, it may take several weeks or even months to recognize the success or failure of therapy using these methods. Furthermore, it is difficult to distinguish a residual tumor from necrosis or fibrosis by stiffness. The serum marker cancer antigen (CA)-125 is frequently elevated at the time of EOC diagnosis and is commonly used to assess the response to treatment. The reliability of the response evaluation during chemotherapy for serous EOC may be improved by the assessment of serum human epididymis protein 4 (4). However, Le *et al* (5) suggested that the CA-125 response to NACT was not significantly predictive of progression-free survival.

Ultrasound elastography is an emerging dynamic imaging technique, which has been established as a promising modality to discriminate relative tissue stiffness by surveying the extent of deformation associated with strain under the utilization of an external pressure. Elastography has been demonstrated to be a useful method for ascertaining the presence of tumors and distinguishing between different categories of abnormalities, such as benign versus malignant lesions in breast tissue, thyroid tissue, prostate tissue and lymph nodes (6). Furthermore, it has been applied for the evaluation of the effect of ablative therapies, in which the heating of tissues results in the denaturation of proteins and consequently promotes the stiffness of ablated tissue (7). Elastography has also been acknowledged as a practical method that can potentially identify clear differences between the responses of malignant tissues, such as breast cancer, to NACT (8).

Accordingly, in the present study, we hypothesized that the evaluation of tumor stiffness by elastography has the potential to provide additional information that is useful in predicting the response to NACT in a clinical setting. To test this hypothesis, the tumor stiffness in patients with advanced EOCs who received NACT was investigated and the correlation with whether optimal cytoreduction could be completed or not was analyzed.

## Materials and methods

### Patients

A total of 32 patients with International Federation of Gynecology and Obstetrics stage III and IV EOC treated with NACT (paclitaxel-platinum combination) and interval cytoreductive surgery at the Department of Gynecologic Oncology, Obstetrics and Gynecology Hospital of Fudan University (Shanghai, China) between January 2011 and December 2012 were selected for enrolment into this study. The study design and protocol were approved by the Institutional Review Board of the Obstetrics and Gynecology Hospital of Fudan University, and all patients provided written informed consent once the nature of the procedure had been explained fully.

### Treatment

In all cases, NACT was administered following confirmation of the cancer diagnosis by cytological or histological examination. Prior to NACT administration, one of the 32 patients was diagnosed histologically by laparoscopy, two were diagnosed by laparotomy and the remaining 29 patients were diagnosed by cytological samples obtained from abdominal paracentesis. During interval cytoreductive surgery, histological confirmation of the disease was also performed in all study patients.

A standard paclitaxel-platinum combination regimen was administered as NACT. Carboplatin was administered with an area under the curve of 5, together with paclitaxel (175 mg/m^2^). All patients were administered only one course of chemotherapy for one day and all patients underwent pelvic sonography, which included transvaginal and transabdominal imaging, followed by pre- and post-NACT (three weeks after NACT) elastography.

All procedures were performed by a single radiologist with 10 years of experience in transvaginal and transabdominal sonography who had specialized in elastography for the last three years. All patients underwent imaging with an Acuson S2000 system (Siemens Medical Solutions, Mountain View, CA, USA) using a transvaginal 7-MHz probe and a transabdominal 4–5-MHz probe. First, with conventional transvaginal and transabdominal sonography, the tumor was located and assessed for its size and overall sonographic appearance. Three vessels were measured and the mean pulsatility and resistive indices were calculated. The section of the ovarian tumor with the largest solid component was selected for analysis. In each scanning plane selected for documentation with conventional sonography, transabdominal elastography was also performed immediately after acquisition of the conventional grayscale sonogram. Next, the real-time elastogram and grayscale sonogram were displayed simultaneously in the dual mode. The region of interest in the elastogram was set to include sufficient surrounding mass tissue. The tissue elasticity information was superimposed over the sonogram and displayed in color, with green indicating medium tissue stiffness, red indicating hard tissue and blue indicating soft tissue, as confirmed by previously published studies that had used the Acuson S2000 system (9).

### Imaging analysis

The elastograms were evaluated using four-point scale from a study of neck masses (Table I) (10,11). Although the machines used in the studies varied, the principle of imaging tissue stiffness was consistent. Due to the lack of universally accepted criteria for scoring elastograms of ovarian cancer in the published literature and the novel nature of this study, for easy analysis of the images, the elastograms were graded on the simplified four-point scale, evaluating the stiffness of the solid areas surrounded by fluid in the mass (Table I). This scale was adapted from a previous thyroid ultrasound elastographic study (11). This scale was used as a standard in our prior study and was found to be feasible for serous ovarian cancer (9).

The elastograms were evaluated on the basis of their conventional sonographic presentation. All data collection and imaging analysis were performed prospectively by another radiologist who also had 10 years of experience in sonography and had specialized in elastography for the last three years. In addition, the radiologist was blinded to the final histological diagnoses. All patients underwent surgery, and the pathological diagnoses were categorized as of ovarian origin based on the conventional criteria of the two pathologists.

Following NACT, all patients underwent interval cytoreductive surgery regardless of their responses to NACT. The optimality was defined according to the Gynecologic Oncology Group definition in which optimal cytoreduction was defined as <1 cm of the maximal residual tumor size (1). The pathological diagnoses were categorized as of ovarian origin based on the conventional criteria of two pathologists post-operatively. Cases not confirmed as high-grade serous ovarian carcinoma (HGSC) were excluded.

### Statistical analysis

The SPSS version 11.0 for Windows software package (SPSS, Inc., Chicago, IL, USA) was used for statistical data analysis. Data are presented as the mean ± standard deviation. Student’s t-test was applied to determine whether tumor sizes, CA-125 levels and resistive and pulsatility indices differed between the pre- and post-NACT groups. Fisher’s exact test was performed to determine whether the elasticity scores varied between the two groups. The diagnostic sensitivity, specificity, accuracy, positive predictive value (PPV) and negative predictive value (NPV) were also calculated.

## Results

Of the 32 patients studied, eight (25%) were excluded by histopathological analysis for diagnoses not pertinent to this study, including case of one fallopian tube cancer, two mucinous carcinomas, two cases of clear cell cancer, one metastatic carcinoma (peritoneum), and two tumors of undetermined origin. The remaining cases (24/32; 75%) were all of HGSC.

The sonographic and serum marker characteristics of the lesions pre- and post-NACT are shown in Table II. No statistically significant differences were identified between the mean tumor diameters and the CA-125 levels pre- and post-NACT. The resistive and pulsatility indices were also not significantly different pre- and post-NACT. However, the mean elasticity score was statistically higher for the post-NACT lesions than for the pre-NACT lesions (3.13±0.57 vs. 2.04±0.51, respectively; P<0.001).

**Figure 1:**
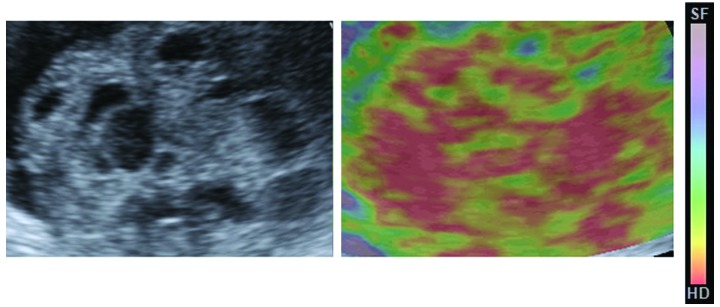
Conventional sonogram and elastogram from a pre-neoadjuvant chemotherapy patient with high-grade serous ovarian carcinoma, which appears predominantly yellow and green, and is clearly distinguishable. The case was scored as 2 on the four-point elasticity scale.

**Figure 2:**
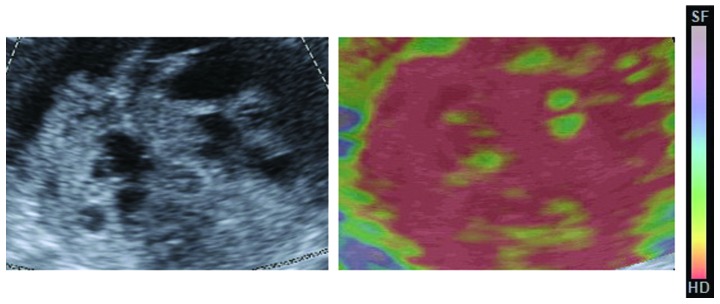
Conventional sonogram and elastogram from the same patient as shown in Fig. 1, but post-NACT, which appears predominantly red and is clearly distinguishable. The case was scored as 4 on the four-point elasticity scale. However, the image from the conventional ultrasound was almost the same as that of the pre-NACT. NACT, neoadjuvant chemotherapy.

The median elasticity score for the pre-NACT lesions on the four-point scale was 2, and the score for the post-NACT lesions was 4. The distributions of the elasticity scores for pre- are post-NACT are shown in Table III. Of the pre-NACT lesions, 79.2% were scored as 1 or 2, and 20.8% were scored as 3. Of the post-NACT lesions, 66.7% were scored as 3 and 4, and 33.3% were scored as 1 and 2 (Figs. 1 and 2). Cases of post-NACT with scores of 3 and 4 had a higher optimal cytoreduction rate than the cases with scores of 1 and 2 (93.8 vs 25.0%, respectively; P<0.001). When the post-NACT elasticity scores of 3 and 4 were used for the prediction of optimal cytoreduction, elastography had 88.2% sensitivity, 85.7% specificity, a 93.8% PPV, a 75.0% NPV and 87.5% accuracy.

## Discussion

EOCs have the highest mortality rates in gynecological oncology, occurring in the advanced stage and having spread beyond the ovary at the time of diagnosis due to its rapid growth. EOCs are rarely detected at an early stage and always exhibit p53, BRCA, WT1 and p16 mutations, as well as high (>10%) Ki67 levels (1). Defined as the chemotherapy modality prior to primary surgery, NACT is an alternative method to primary optimal cytoreduction for the management of advanced EOC (12). The most significant potential benefit of NACT is increasing the percentage of patients who are subsequent candidates for achieving optimal cytoreduction. In patients with advanced ovarian cancer, optimal cytoreduction can be selected by evaluating the response to NACT by ultrasound, CT, MRI and PET, as well as by analysis of the levels of CA-125 (13).

Elastography, a novel functional imaging technique, may allow the earlier identification of a response, with higher accuracy. At present, the main role of elastography is to discriminate between cancer and benign lesions associated with conventional ultrasonography and to decrease the administration of unnecessary puncture biopsies, which is based on the tissue stiffness (14). The diagnosis depends on the principle that cancer is relatively hard compared with non-cancerous lesions, however, relatively soft cancers do also exist.

Hayashi *et al* (15) reported that the mean breast elastography scores were significantly lower for the clinical and pathologic complete response groups than for the other groups. Tumor stiffness assessed by elastography was potentially predictive for the response to NACT. Evaluated by elastography, tissue stiffness may be confirmed as a clinically significant tumor feature, which cannot be gained by other functional imaging techniques (15). Similarly, in an additional study, Falou *et al* (8) identified an early clinically response in patients during the process of NACT by elastography. These results indicated that elastography may potentially be applied as an early predictor of tumor treatment response in patients with cancer (8).

The present study demonstrated that elastography may be useful in predicting the outcome of neoadjuvant therapy in patients with advanced HGSC. In our prior study (9), two different types of serous ovarian cancer were evaluated that showed different stiffness ranges on elastography. A tendency for HGSC to be less stiff than low-grade serous carcinoma (LGSC) was identified, which may be elucidated by the fact that the predominantly solid tissue of HGSC developed necrosis rapidly. Conversely, LGSC grew relatively slowly so that its solid lesions were less flexible and more stiff (9). Recent studies have confirmed that chemotherapy based on platinum for patients with the p53 mutation has higher sensitivity, in contrast to previous theories (16,17). Indeed, owing to the loss of the capability to repair DNA, rapidly proliferating cells of HGSC are more sensitive to platinum (16). Chemotherapy has been found to control the development of cell necrosis in force, and the stiffness of HGSC post-NACT is likely to develop toward that of LGSC (17). As a result, the mean elasticity score of the pre-NACT lesions was lower than that of the post-NACT lesions in the present study. This indicated that techniques with functions rather than anatomy imaging may provide improved precision in monitoring response, as functional changes are predicted to develop prior to morphological changes.

In the current study, it was also confirmed that cases with scores of 3 and 4 post-NACT had higher optimal cytoreduction rates than cases with scores of 1 and 2. Since all patients were administered only one course of chemotherapy, this confirmed that elastography is a sensitive tool for the evaluation of NACT in patients with HGSC.

There were certain limitations to the current study. First, the sample size was small, and the aim of this study was not to compare elastography against conventional sonography, CT and MRI, but to evaluate its potential role as a novel tool. Furthermore, due to a lack of universally accepted criteria for scoring elastograms of ovarian cancer in the published literature, a simplified four-point scale was used based on a previous thyroid elastographic study (11) similar to our prior study (9). In addition, the section of the ovarian tumor with the largest solid component was selected for analysis and did not represent all of the lesions. Finally, all the patients exhibited HGSCs, and further investigation is required to analyze whether the results can be applied to other EOCs.

To the best of our knowledge, this is the first study to investigate the application of elastography in the evaluation of NACT in HGSC. Despite its small sample size and retrospective design, several conclusions can be drawn from the study. First, elastography is a more sensitive tool for the evaluation of NACT in patients with HGSC compared with CT and MRI. Furthermore, elastography may aid gynecologists in choosing the optimal cytoreduction. We hypothesize that elastography is likely to become a standard tool for the assessment of the treatment response to NACT in HGSC based on the results of future studies.

## Figures and Tables

**Table I:** Elasticity scoring systems for cervical lymph nodes.

Elastographic score^a^	Elastographic appearance
1 (soft)	Predominantly purple, green or yellow, with <10% displaying red. The node is indistinguishable from the surrounding tissues
2 (moderately soft)	Predominantly yellow or green, with red areas comprising between 10 and 50%. The node is partially delineated from the surrounding tissues
3 (moderately stiff)	Predominantly red, with yellow or green areas comprising between 10 and 50%. The node is partially delineated from the surrounding tissues
4 (stiff)	Predominantly red, with <10% appearing yellow or green. The node is distinguishable from the surrounding tissues

a Adapted from a previous thyroid elastographic study (11).

**Table II:** Characterization of pre- and post-NACT using conventional sonography and serum marker.

Index	Pre-NACT	Post-NACT	P-value
Mean diameter (cm)	10.27±6.56	11.56±5.22	0.61
CA-125 (U/ml)	675.32±87.78	703.96±79.81	0.76
Resistance index	0.34±0.24	0.31±0.34	0.57
Pulsatility index	0.82±0.43	0.76±0.35	0.71
Mean elasticity score	2.04±0.51	3.13±0.57	<0.001

Data are presented as the mean ± standard deviation. NACT, neoadjuvant chemotherapy; CA-125, cancer antigen 125.

**Table III:** Elasticity scores in pre- and post-NACT lesions (n=24).

Elasticity score	Pre-NACT, n	Post-NACT, n	Optimal cytoreduction, n (%)
1	4	2	0
2	15	6	2 (25.0)^a^
3	5	3	3
4	0	13	12 (93.8)^b^
Total	24	24	17

Scores

a 1 and 2; and

b 3 and 4.

NACT, neoadjuvant chemotherapy.

## Abbreviations

NACT: neoadjuvant chemotherapy
HGSC: high-grade serous ovarian carcinoma
CA-125: cancer antigen 125
NPV: negative predictive value
PPV: positive predictive value
EOC: epithelial ovarian cancer
